# Epidemiology of Enterotoxigenic *Escherichia coli* infection in Minnesota, 2016–2017

**DOI:** 10.1017/S0950268820001934

**Published:** 2020-09-01

**Authors:** S. Buuck, K. Smith, R. C. Fowler, E. Cebelinski, V. Lappi, D. Boxrud, C. Medus

**Affiliations:** 1 Foodborne, Waterborne, Vectorborne, and Zoonotic Diseases Section, Minnesota Department of Health, Saint Paul, MN, USA; 2 Public Health Laboratory, Minnesota Department of Health, Saint Paul, MN, USA; 3 Laboratory Leadership Service, Centers for Disease Control and Prevention, Atlanta, GA, USA

**Keywords:** Diarrhoea, Enteric bacteria, *Escherichia coli* (*E. coli*), food-borne infections

## Abstract

Enterotoxigenic *Escherichia coli* (ETEC) is a well-established cause of traveller's diarrhoea and occasional domestic foodborne illness outbreaks in the USA. Although ETEC are not detected by conventional stool culture methods used in clinical laboratories, syndromic culture-independent diagnostic tests (CIDTs) capable of detecting ETEC have become increasingly prevalent in the last decade. This study describes the epidemiology of ETEC infections reported to the Minnesota Department of Health (MDH) during 2016–2017. ETEC-positive stool specimens were submitted to MDH to confirm the presence of ETEC DNA by polymerase chain reaction (PCR). Cases were interviewed to ascertain illness and exposures. Contemporaneous *Salmonella* cases were used as a comparison group in a case-case comparison analysis of risk factors. Of 222 ETEC-positive specimens received by MDH, 108 (49%) were concordant by PCR. ETEC was the sixth most frequently reported bacterial enteric pathogen among a subset of CIDT-positive specimens. Sixty-nine (64%) laboratory-confirmed cases had an additional pathogen codetected with ETEC, including enteroaggregative *E. coli* (*n* = 40) and enteropathogenic *E. coli* (*n* = 39). Although travel is a risk factor for ETEC infection, only 43% of cases travelled internationally, providing evidence for ETEC as an underestimated source of domestically acquired enteric illness in the USA.

## Background

Enterotoxigenic *Escherichia coli* (ETEC) infection is characterised by watery diarrhoea that typically lasts 3–5 days; illness can range from mild and self-limiting to cholera-like. It is a major cause of diarrhoea among children living in developing countries, as well as among travellers to developing countries [1]. ETEC is transmitted through the faecal–oral route, most commonly through contaminated food (especially produce) or water [2].

ETEC strains cause illness through the production of heat-labile enterotoxin (LT) or heat-stable enterotoxin (ST), which disrupt intestinal function leading to profuse, watery diarrhoea. Symptoms are often more severe when ST is present (either alone or with LT), compared with the presence of LT alone [3].

In countries where ETEC is endemic, children are often infected multiple times early in life and subsequently develop immunity. Globally, there are an estimated 220 000 000 cases and over 50 000 deaths annually, primarily occurring in developing countries among children aged <5 years [4]. In developed countries, ETEC infection has been associated mostly with traveller's diarrhoea among visitors to endemic developing countries, causing an estimated 10 000 000 cases of traveller's diarrhoea annually [5]. In the USA, an estimated 40 000 ETEC cases occur annually, with 55% associated with international travel and 45% acquired domestically through foodborne transmission [6].

ETEC is not detected by conventional stool culture methods used in clinical laboratories. However, public health investigations have used alternative methods to identify ETEC as the aetiology for occasional foodborne and waterborne illness outbreaks in the USA [7, 8, 9]. Furthermore, sentinel surveillance conducted at two clinical laboratories in Minnesota during 2000–2008 identified ETEC as the second and fourth most common reportable enteric bacterial pathogen, and 39% of identified cases were domestically acquired [10].

Since 2015, clinical laboratories have increasingly used commercial culture-independent diagnostic tests (CIDTs), particularly multiplex polymerase chain reaction (PCR) syndrome panels [11, 12], for routine detection of enteric pathogens, making it possible to detect ETEC and other diarrhoeagenic *E. coli* pathotypes. From late 2015 to 2017, a total of 17 clinical laboratories in Minnesota adopted the FilmArray^®^ gastrointestinal panel (GIP) (BioFire^®^ Diagnostics, Salt Lake City, UT), which detects 22 enteric pathogens, including ETEC and other diarrhoeagenic *E. coli* pathotypes (Shiga toxin-producing *E. coli* (STEC), enteroaggregative *E. coli* (EAEC), enteroinvasive *E. coli* (EIEC) and enteropathogenic *E. coli* (EPEC)). Minnesota rules require clinical laboratories to report and submit ETEC-positive clinical samples to the state public health laboratory. The uptake of the FilmArray^®^ GIP and detailed surveillance interviews conducted with cases combined to provide a unique opportunity to assess the epidemiology and public health importance of ETEC infections in Minnesota.

## Methods

This study describes the clinical and epidemiological characteristics of ETEC infections in Minnesota using data collected as part of routine laboratory-based surveillance for ETEC conducted from 1 January 2016 to 31 December 2017. All diarrhoeagenic *E. coli*, including ETEC, are reportable pathogens in Minnesota, and clinical laboratories are required to submit an isolate or specimen from positive clinical samples to the Minnesota Department of Health (MDH) Public Health Laboratory (PHL). As part of Minnesota's reportable disease rules, health care providers reported patient names, contact information, hospitalisation and outcome (i.e. alive or deceased). An ETEC case was defined as a Minnesota resident who had an ETEC-positive stool specimen detected either at a clinical laboratory or at the MDH PHL. Confirmed cases were those that had LT, ST or both toxin genes detected by PCR at the MDH PHL. Probable cases were those with no LT or ST toxin gene detected by PCR at the MDH PHL. Cases whose clinical materials were not received at the MDH PHL were excluded from analyses comparing confirmed and probable cases, but included in analyses of all cases.

### Epidemiological data collection

MDH staff attempted to contact each case to conduct a detailed surveillance interview regarding illness, demographics, international travel and possible exposures during the week before illness onset. The period of 1 week before illness onset was chosen to align with the standard data collection instruments in use for routine surveillance of other enteric pathogens. Cases who did not travel before illness onset were asked all questions on the questionnaire, and cases who travelled were asked a subset of questions, including travel destination, attendance at a daycare or preschool, occupation and race/ethnicity. Cases were interviewed regardless of whether their illness began while travelling or after returning to the USA.

Case reports sent from clinical laboratories to MDH also noted any other reportable enteric pathogens that were detected in ETEC-positive stool specimens. For a subset of cases, clinical laboratories also provided complete FilmArray GIP results, including results for pathogens that are commonly detected but not mandatorily reportable in Minnesota, such as *Clostridioides difficile*, norovirus and rotavirus.

At the start of the study, five clinical laboratories were performing the FilmArray GIP, with an additional 12 clinical laboratories adopting this testing platform by the end of the study period. Test results from the initial five clinical laboratories were used to determine the frequency of ETEC detection, compared with other reportable enteric pathogens, and ETEC seasonality.

### Laboratory testing

At the MDH PHL, ETEC-positive stool specimens were inoculated on Sorbitol MacConkey agar, and crude nucleic acid extracts were prepared from culture growth as previously described [10]. Crude extracts were tested for LT and ST toxin genes using real-time PCR. ETEC PCR was conducted in a final volume of 25 μl with 1x SYBR GreenER qPCR SuperMix, 1 μl of crude extract, and previously published *estA* and *eltB* primer sets [10]. Amplification conditions were the same as described by Medus *et al*.; however, interpretations of melt curve temperatures were slightly different because of changes in mastermix components. PCR was considered positive for ETEC when there was a melt curve for *estA* (Tm = 73 ± 0.5 °C) or *eltB* (Tm = 77 ± 0.5 °C).

### Statistical analyses

Statistical analyses were performed using SAS^®^ Version 9.4 (SAS Institute, Inc., North Carolina, USA). Fisher's exact test (two-tailed) was used to compare demographic characteristics and prevalence of specific symptoms between confirmed and probable ETEC cases. To describe domestically acquired ETEC infections, cases were also stratified based on international travel during the week before illness onset, and a comparison between travellers and non-travellers was made on the same demographic and symptom variables. The threshold for statistical significance was set at *α* = 0.05 for all analyses.

A univariate analysis compared exposures of confirmed ETEC cases to exposures of *Salmonella* cases confirmed by culture at the MDH PHL from the same study period. *Salmonella* cases associated with outbreaks and/or international travel were excluded from the analysis. In addition to the 51 food items on the food consumption history section of the questionnaire, other risk factors included in this analysis were eating at a restaurant, contact with animals, swimming, contact with someone experiencing diarrhoeal illness, and living on, working on or visiting a farm with animals. Cases who could not be reached or who declined to be interviewed were excluded from these analyses, but were included in analyses of demographic and laboratory results. Mantel-Haenzel odds ratios were calculated to assess differences in exposure to different risk factors and specific food items between ETEC and *Salmonella* cases.

## Results

### Laboratory characteristics

In total, 244 cases were identified. Nearly all cases tested positive for ETEC at clinical laboratories that used the FilmArray GIP (*n* = 240, 98.4%). Additionally, two cases (0.8%) initially tested positive for STEC by the VERIGENE^®^ Enteric Panel (Luminex Corporation, Austin, Texas), but were later identified as having ETEC at the MDH PHL. One case (0.4%) was identified at MDH after submitting a stool sample for testing directly to the PHL. One case (0.4%) was tested at an out-of-state clinical laboratory by an unknown testing method. Of 244 ETEC cases, the MDH PHL received ETEC-positive stool specimens from clinical laboratories for 222 cases (91%) and confirmed the presence of ETEC in 49% (*n* = 108). Among confirmed cases, both ST and LT enterotoxin genes were detected in 30 cases (28%), ST only in 58 (54%) and LT only in 20 (19%).

### Demographic characteristics

Among all confirmed and probable cases, 52% of cases were male (Table 1). Those who self-reported their race as white accounted for 85% of cases, with 11% reporting Hispanic ethnicity. Median age of cases was 44 years, with 15% of cases aged <18 years. A higher proportion of confirmed cases than probable cases reported Hispanic ethnicity (16% *vs.* 6%; *P* = 0.04); otherwise, demographic variables of confirmed cases did not significantly differ from those of probable cases (Table 1). Compared with estimates of the entire Minnesota population, a higher proportion of ETEC cases reported Hispanic ethnicity and a lower proportion was aged <18 years [13].

**Table 1.** Demographics by case status, ETEC cases reported in Minnesota 2016–2017

	Confirmed cases (*n* = 108)	Probable cases (*n* = 114)	All cases (*n* = 222)	2017 Minnesota population (estimated)[12]
Male (%)	56	48	52	50
White (%)	86	84	85	86
Hispanic (%)	16^a^	6	11	5
Median age (yrs)	42.5	46.5	45	37
Aged <18 years (%)	14	17	15	23
Travelled internationally (%)	43^a^	22	36	n/a

a Significantly different from probable cases at the *P* < 0.05 level.

### Illness characteristics

Diarrhoea was the most commonly self-reported symptom (96% of those interviewed), which lasted a median of 8 days (Table 2). Cramps were reported by a majority of cases (79%); less commonly reported symptoms included fever (42%), headache (38%), vomiting (28%) and bloody stools (21%). Fifteen per cent of cases were hospitalised, but no cases died. Probable cases more frequently reported vomiting and bloody stools than confirmed cases (34% *vs.* 20%; *P* = 0.04 (vomiting), and 31% *vs.* 12%; *P* = 0.005 (bloody stools)). Symptoms and hospitalisation rate did not differ significantly between cases for whom ETEC was the only pathogen detected and those who had at least one other pathogen detected.

**Table 2.** Symptoms by codetection of other pathogens at clinical laboratory, ETEC cases reported in Minnesota 2016–2017

	Confirmed cases (*n* = 108)			Probable cases (*n* = 114)			All cases (*n* = 222)		
	ETEC only detected at clinical laboratory (*n* = 39)	At least one other pathogen detected (*n* = 69)	Total	ETEC only detected at clinical laboratory (*n* = 39)	At least one other pathogen detected (*n* = 75)	Total	ETEC only detected at clinical laboratory (*n* = 78)	At least one other pathogen detected (*n* = 144)	Total
Diarrhoea (%)	97	98	98	96	97	97	97	97	97
Vomiting (%)	19	20	20^a^	37	31	34	28	25	27
Bloody stools (%)	13	12	12^a^	26	32	31	19	22	21
Cramps (%)	81	82	82	78	81	80	80	81	81
Fever (%)	40	40	40	38	45	43	39	42	41
Headache (%)	55	35	42	19	37	31	38	36	36
Median stools per 24 h	10	8	9	10	9	10	10	9	10
Median duration of diarrhoea (days)	8.5	8	8	14	7	7	10	8	8
Hospitalised (%)	13	9	10	5^b^	21	17	9	15	14

a Significantly different from probable cases at the *P* < 0.05 level.

b Significantly different from probable cases with codetected pathogens at the *P* < 0.05 level.

### International travel

International travel data were available for 204 of 244 cases. Sixty-six cases (36%) reported travelling to another country during the week before illness onset (Table 1). More confirmed cases reported international travel than probable cases (43% *vs.* 22%; *P* = 0.003). Symptoms and hospitalisation rate did not differ significantly between international travellers and non-travellers (data not shown). Overall, cases travelled to 41 countries across different regions of the world; Mexico was the most frequent destination for travellers, accounting for 21 cases (32% of travellers) (Table 3).

**Table 3.** Travel locations for cases who travelled internationally, ETEC cases reported in Minnesota 2016–2017

Country	Confirmed cases	Probable cases	All cases
Mexico	14	7	21
Dominican Republic	4	4	8
Guatemala	2	1	3
India	2	1	3
Nicaragua	3	0	3
Vietnam	2	1	3
Cuba	1	1	2
Egypt	1	1	2
France	1	1	2
Haiti	1	1	2
Kenya	1	1	2
Somalia	0	2	2
29 other countries	–	–	1 each

### Comparison with other reportable enteric pathogens

MDH received 1781 case reports for enteric pathogens from five clinical laboratories that used the FilmArray GIP for the entire study period. ETEC accounted for 8.5% of reportable enteric pathogens (*n* = 152), making it the sixth most commonly reported enteric pathogen following *Campylobacter* spp. (25.9%; *n* = 462), EAEC (20.8%; *n* = 370), EPEC (13.5%; *n* = 240), *Salmonella* spp. (11.3%; *n* = 201) and STEC (11.2%; *n* = 199). Enteric bacterial pathogens reported less commonly than ETEC included *Shigella* spp. (5.0%; *n* = 89), *Yersinia enterocolitica* (2.8%; *n* = 50) and *Vibrio* spp. (1.0%; *n* = 18).

### Codetections

Additional reportable enteric pathogens were frequently identified in ETEC-positive stool specimens. In total, 145 (65%) ETEC cases had at least one other reportable enteric pathogen detected, accounting for 69 (64%) of 108 confirmed ETEC cases and 76 (67%) of 114 probable ETEC cases (Table 4). Of these, 59 (41%) had at least two reportable enteric pathogens detected in addition to ETEC, and nine (6%) had three or more other reportable enteric pathogens detected. EPEC (*n* = 77) and EAEC (*n* = 72) were the most commonly codetected pathogens, followed by STEC (*n* = 32), *Campylobacter* spp. (*n* = 15), *Shigella*/EIEC (*n* = 14), *Salmonella* spp. (*n* = 11), *Vibrio* spp. (*n* = 3), *Cryptosporidium* spp. (*n* = 2) and *Y. enterocolitica* (*n* = 1).

**Table 4.** Additional pathogens codetected at clinical laboratories in ETEC-positive specimens, ETEC cases reported in Minnesota 2016–2017

Pathogen	Number detected (%) in confirmed ETEC cases (*n* = 108)	Number detected (%) in probable ETEC cases (*n* = 114)	Number detected (%) in all ETEC cases (*n* = 222)
At least one other pathogen	69 (64%)	76 (67%)	145 (65%)
At least two other pathogens	29 (27%)	30 (26%)	59 (27%)
EPEC	39	38	77
EAEC	40	32	72
STEC non-O157	7	19	26
*Campylobacter*	4	11	15
EIEC/*Shigella*	7	7	14
*Salmonella*	1	10	11
STEC O157	0	6	6
*Vibrio*	1	2	3
*Cryptosporidium*	0	2	2
*Yersinia*	1	0	1

EPEC, enteropathogenic *E. coli*; EAEC, enteroaggregative *E. coli*; STEC, Shiga toxin-producing *E. coli*; EIEC, enteroinvasive *E. coli*.

Among cases who travelled internationally, 91% (60 of 66) had additional codetected pathogens, compared with 53% (73 of 138) of those who did not report international travel (*P* < 0.001). EAEC (68%; *n* = 45) and EPEC (50%; *n* = 33) were the most frequently codetected pathogens among international travellers; in 38% (*n* = 25) of travel-associated ETEC cases, both EAEC and EPEC were also detected. Among non-travellers, EPEC (22%; *n* = 30) was the most commonly codetected pathogen, followed by EAEC (14%; *n* = 19) and STEC non-O157 (12%; *n* = 17).

Complete FilmArray GIP results that included reportable and non-reportable pathogens were received for a subset of 93 ETEC cases. In 62 (67%) cases, another pathogen was detected. Of these, 20 (22%) had a non-reportable pathogen detected, including *C. difficile* (*n* = 10), norovirus (*n* = 6), *Plesiomonas shigelloides* (*n* = 2), adenovirus (*n* = 1), astrovirus (*n* = 1), rotavirus (*n* = 1) and sapovirus (*n* = 1).

### Seasonality

**Fig. 1.**
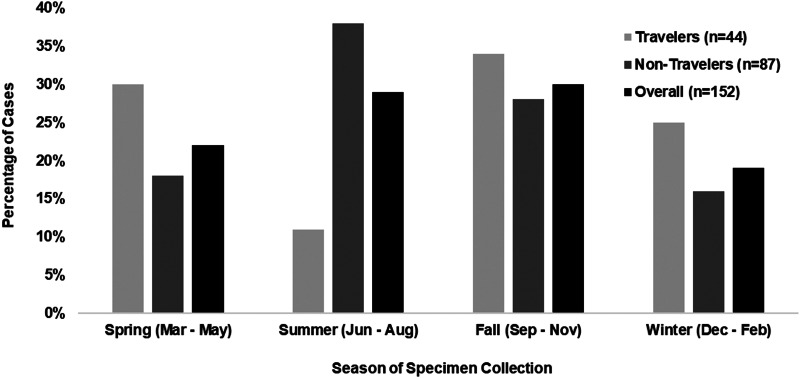
Percentage of ETEC-positive stool specimens reported to the Minnesota Department of Health (MDH) during each season, with cases' history of international travel within the week before illness onset. Total number of cases includes only those clinical laboratories using the FilmArray GIP during the entire study period. Seasons were defined as follows: spring, March–May; summer, June–August; fall, September–November; and winter, December–February. Information on travel status was not available for 21 (14%) cases. However, these cases are included in the Overall column for each season.

Seasonality analysis was restricted to 152 ETEC cases reported by the five clinical laboratories using the FilmArray GIP during the entire study period. The most frequently reported season of infection was fall (*n* = 45; 30%), followed by summer (*n* = 44; 29%), spring (*n* = 34; 22%) and winter (*n* = 29; 19%) (Fig. 1). Cases who travelled internationally (*n* = 44) had limited seasonal variation. Among non-travellers (*n* = 87), seasonality was more prominent, with 57 (66%) cases occurring in either summer or fall.

### ETEC and *Salmonella* case-case comparison

Compared with sporadic, domestically acquired *Salmonella* cases from the same period, domestically acquired ETEC cases were significantly more likely to report consumption of celery, cucumbers, sesame seeds, citrus fruits other than oranges, and melon other than watermelon or cantaloupe (Table 5). ETEC cases were significantly less likely to report contact with animals, eating at restaurants, consumption of spinach and consumption of lettuce other than iceberg or romaine.

**Table 5.** Case-case comparison of confirmed ETEC and confirmed *Salmonella* cases from 2016 to 2017, selected exposures

	Confirmed ETEC cases (*n* = 108)	Confirmed *Salmonella* cases (*n* = 964)	
Reported exposure	No. (%)	No. (%)	Odds ratio (95% CI)
Celery	12 (27)	117 (13)	2.45 (1.2–4.9)
Cucumbers	24 (49)	242 (28)	2.52 (1.4–4.5)
Citrus other than oranges	15 (30)	149 (17)	2.15 (1.2–4.0)
Melon other than watermelon or cantaloupe	3 (19)	46 (5)	4.34 (1.2–15.8)
Sesame seeds	5 (10)	27 (3)	3.60 (1.3–9.8)
Animal contact	25 (47)	607 (64)	0.50 (0.3–0.9)
Eating at a restaurant	33 (59)	668 (72)	0.55 (0.3–1.0)
Lettuce other than iceberg or romaine	1 (2)	109 (12)	0.15 (0.0–1.1)
Spinach	4 (9)	190 (22)	0.35 (0.1–1.0)

## Discussion

This study adds to a growing body of evidence indicating that ETEC is an underestimated aetiology of enteric illness in the USA. Among clinical laboratories in Minnesota using the FilmArray GIP during 2016–2017, ETEC was the sixth most commonly reported bacterial enteric pathogen and detected in 8.9% of specimens that tested positive for at least one reportable pathogen. Other laboratories that used CIDTs have reported ETEC detection rates ranging from 3.5% to 4.7% among all CIDT-positive specimens [11, 12, 14]. Before the use of CIDTs, sentinel surveillance in Minnesota during 2000–2008 demonstrated that ETEC was the second (1.9% of stools submitted for bacterial culture) and fourth (0.8%) most commonly reported bacterial enteric pathogen from two clinical laboratories [10]. However, that study did not include EAEC and EPEC, which were both detected more frequently than ETEC in this study. Without CIDTs, most clinical laboratories cannot test for ETEC; this study demonstrates that when CIDTs capable of detecting ETEC are used, cases are frequently identified.

ETEC has long been recognised as a major cause of traveller's diarrhoea, but an important finding of this study was that domestically acquired ETEC infections made up a substantial proportion of all cases; 68% of ETEC cases (57% among confirmed cases) reported no travel outside of the USA in the week before illness onset. In comparison, a previous study reported that 39% of ETEC cases identified in Minnesota during 2000–2008 did not travel internationally [10]. Because cases were only asked about the 7 days prior to illness onset, it is possible that cases with incubation periods longer than 7 days were misclassified as domestically acquired. However, this likely represents a small proportion of cases and the previous study used the same methodology to identify travel-associated cases.

The case-case comparison study of ETEC and *Salmonella* cases identified foods that were more likely to be consumed by ETEC cases, including several produce items. Imported produce items have been implicated as the source of occasional ETEC outbreaks in the USA and other developed countries [8, 15], and represent a plausible source of sporadic, domestically acquired ETEC infections as well. No outbreaks were identified during the study period, but it is likely that more outbreaks will be identified in the future as more cases are identified by CIDTs and more states adopt ETEC surveillance. However, further evidence is required to determine which ETEC infections are clinically significant, especially when codetected with other enteric pathogens.

There was not a marked seasonality among cases who travelled internationally; however, domestically acquired cases primarily occurred during the summer and fall months. Increased rate of ETEC infection among travellers to endemic countries has been associated with increased ambient temperature, suggesting a seasonal effect with more cases occurring during warmer months [16].

Symptom profiles of ETEC cases were similar to those reported in literature summarizing ETEC outbreaks in the USA [7, 9]. Nearly all cases in this study reported diarrhoea (96%), with fewer cases reporting fever (42%) and vomiting (28%). Bloody stools, an uncommonly reported symptom of ETEC infection, were reported by a significantly higher proportion of probable cases (31%), compared with confirmed cases (12%). Some of this difference could be explained by the more frequent presence of pathogens that commonly cause bloody stools among probable cases; for example, STEC was codetected in 18% of probable ETEC cases compared to just 6% of confirmed cases. Additionally, it is possible that some cases could have misidentified the presence of blood in stools. This finding indicates that illness among some probable ETEC cases may have been caused by pathogens other than ETEC.

Symptoms did not differ between travellers and non-travellers, nor between those with and without other codetected pathogens. It is possible that the lack of significant differences between these groups was due to a lack of power, given the limited sample sizes of certain categories.

Cases reported diarrhoea lasting a median of 8 days, which is longer than has previously been reported [1]. The longer duration of illness could be the result of bias in the population of ETEC infections included in this study. As cases in this study were all found to be sporadic rather than outbreak-associated, it is possible that cases with longer duration of diarrhoea were more likely to seek medical care and be tested for enteric pathogens, and thus more likely to be included in the study population.

Both ST and LT enterotoxin genes were detected in 28% of confirmed cases, ST alone in 54% and LT alone in 19%. This is a slightly higher proportion of ST-only cases than reported by Isidean *et al*., although the authors noted considerable variation at different study sites worldwide [17].

Codetections with other reportable pathogens were identified in 64% of ETEC cases, occurring more frequently among international travellers (91% of cases) than domestically acquired cases (53%). Similarly, clinical laboratories in other parts of the USA have previously reported that 78–84% of ETEC-positive specimens were also positive for at least one other pathogen [12, 14]. In our study, the majority of codetections were with other diarrhoeagenic *E. coli* pathotypes, particularly EAEC and EPEC. When multiple pathogens are detected, it is difficult to determine the clinical significance of each pathogen. However, ETEC is a well-documented cause of domestic foodborne illness outbreaks in the USA, and for some proportion of cases, ETEC can reasonably be considered clinically significant. Other diarrhoeagenic *E. coli* such as EAEC and the typical form of EPEC are also recognised as important causes of gastrointestinal illness, especially in developing countries [14, 18–20]. However, the FilmArray GIP does not distinguish between typical EPEC and atypical EPEC, which likely includes pathogenic and non-pathogenic organisms [20]. Because atypical EPEC are much more common than typical EPEC, it is likely that some of the codetected EPEC in this study are not actually pathogenic. True coinfection with ETEC and other diarrhoeagenic *E. coli* pathotypes is plausible, especially in international travel-associated ETEC cases, but additional data are needed to determine the clinical significance of codetections of diarrhoeagenic *E. coli* with other enteric pathogens, particularly for domestically acquired cases.

Only 49% of ETEC CIDT-positive stools at clinical laboratories had concordant results by PCR testing at the MDH PHL; this low concordance could be attributable to factors related to initial detection by the FilmArray GIP, PCR testing used at the MDH PHL, or specimen storage or transport issues that might have affected testing at the MDH PHL. The FilmArray GIP is thought to have increased sensitivity because it uses nested PCR directly on stool specimens. Although this study applied a published method to test for ETEC in CIDT-positive specimens [10], it relies on the detection of ETEC by PCR from viable organisms grown in culture at the MDH PHL rather than detection of ETEC DNA directly from specimens. Therefore, it is possible that a low abundance or presence of non-viable ETEC in the specimen might not be detected by PCR testing. Conversely, a proportion of ETEC-positive CIDT specimens might represent false-positive results because of cross-reactivity with commensal organisms, such as *Citrobacter koseri* and *Hafnia alvei* [14]. Further investigation is needed to resolve discordance between CIDT and reflex PCR testing results as more laboratories adopt CIDTs and begin to routinely diagnose ETEC.

Several clinical laboratories adopted the FilmArray GIP after the start of the study period, meaning detection of ETEC was not possible at these laboratories for at least a portion of the study period. Analyses of seasonality and the frequency of ETEC detection relative to other pathogens were by necessity limited to the subset of cases reported by the five laboratories that were using the FilmArray GIP before the start of the study period. Although these five laboratories were in a range of urban and rural locations around the state, these findings might lack representativeness.

## Conclusion

This is the first study describing the epidemiology of sporadic ETEC infections identified through systematic, routine surveillance in the USA. Although international travel is well established as a risk factor, a majority of infections in this study were domestically acquired, providing evidence that ETEC infection is an underestimated source of enteric illness acquired in the USA.

Further study that requires both laboratory and epidemiological efforts is needed to identify exposures that represent possible sources of domestically acquired ETEC infection. Although the adoption of CIDTs by clinical laboratories is rapidly increasing, use of CIDTs (particularly use of testing platforms that detect ETEC) is not universal and reporting of ETEC-positive stool specimens is not mandatory in most jurisdictions. Therefore, many cases go undetected and the incidence of ETEC infection likely is substantially underestimated. Continuing to develop and incorporate techniques for routine testing and collection of exposure data for cases of ETEC infection will enhance efforts to identify outbreaks and understand the public health importance of ETEC infections in the USA.

## Data Availability

Data available on request due to privacy/ethical restrictions. The data that support the findings of this study are available on request from the corresponding author, SB. The data are not publicly available due to containing private identifying information on individuals, but a redacted copy can be provided on request.
